# A Rare Case of Autoimmune Hemolytic Anemia and Suspected Acute Hemorrhagic Disseminated Encephalomyelitis Following Recent Vaccination

**DOI:** 10.7759/cureus.83556

**Published:** 2025-05-06

**Authors:** Hettiyadura Apsara Surangani Mendis Karunaratne, Karunakalage Anya A De Silva

**Affiliations:** 1 Department of General Medicine, North West Regional Hospital, Burnie, AUS

**Keywords:** acute haemorrhagic disseminated encephalomyelitis, autoimmune hemolytic anemia, encephalitis, vaccination, varicella

## Abstract

Autoimmune hemolytic anemia (AIHA) and acute disseminated encephalomyelitis (ADEM) are rare autoimmune complications that may follow infections or vaccinations. We present a case of a 77-year-old male patient who developed warm AIHA and rapid neurological decline suggestive of hemorrhagic ADEM following recent varicella and influenza vaccinations. Laboratory findings confirmed AIHA, and MRI revealed multifocal white matter lesions with hemorrhagic foci. Despite high-dose corticosteroid treatment, the patient's condition worsened, and he ultimately passed away.

## Introduction

Autoimmune hemolytic anemia (AIHA) is a rare disorder caused by the immune-mediated destruction of red blood cells. It is typically classified based on the thermal reactivity of autoantibodies. Warm AIHA, usually mediated by IgG antibodies, leads to extravascular hemolysis, primarily in the spleen. Common triggers include lymphoproliferative disorders, autoimmune diseases, infections, and, less commonly, vaccinations.

Acute disseminated encephalomyelitis (ADEM) is an uncommon, monophasic inflammatory demyelinating disease of the central nervous system (CNS). It occurs more frequently in children and is rarely reported in adults. ADEM often follows viral infections or, less commonly, vaccinations and is characterized by multifocal neurological deficits and radiologic evidence of diffuse demyelination. Severe variants, such as acute hemorrhagic leukoencephalitis (AHLE), involve hemorrhagic transformation and rapid deterioration.

Although rare, AIHA and ADEM have been independently documented as post-vaccination phenomena. However, their concurrent occurrence following vaccination, particularly in elderly patients, is exceedingly rare. This case highlights a complex clinical presentation of concurrent AIHA and suspected hemorrhagic ADEM after varicella and influenza vaccinations, emphasizing the need for early recognition, comprehensive evaluation, and consideration of vaccine-induced immune dysregulation in elderly patients.

This case also underscores the importance of recognizing rare vaccine-associated autoimmune phenomena, particularly in elderly individuals with potential immune dysregulation. Proposed mechanisms for vaccine-induced autoimmunity include molecular mimicry, bystander activation, and polyclonal B-cell stimulation, particularly relevant in individuals with age-related immune dysregulation.

## Case presentation

A 77-year-old Caucasian male patient presented with a fall and increasing lethargy over the preceding week, without other overt symptoms. His medical history included long-term treatment for hypercholesterolemia (ezetimibe, atorvastatin), hypertension (irbesartan, hydrochlorothiazide), and chronic obstructive pulmonary disease (COPD) (beclomethasone, formoterol, glycopyrronium inhalers). The patient had no recent antibiotic use or history of blood transfusion. Notably, he had received varicella and influenza vaccines 4 and 11 days prior to admission, respectively.

On presentation, the patient was hemodynamically stable and afebrile but exhibited pallor, jaundice, and subtle left facial droop with ataxia. Initial investigations revealed normocytic anemia with a significant drop in hemoglobin from 106 to 96 g/L within 24 hours, an elevated reticulocyte count (186 × 10^^9^/L), and increased total bilirubin with low haptoglobin. The blood smear demonstrated spherocytes and polychromasia and a positive direct antiglobulin test (DAT) for IgG, confirming AIHA (Tables 1, 2).

**Table 1:** Other blood investigations PCR: polymerase chain reaction, CRP: C-reactive protein, LDH: lactate dehydrogenase, EBV: Epstein-Barr virus, CMV: *Cytomegalovirus,* IgG: immunoglobulin G, IgM: immunoglobulin M, INR: international normalized ratio, APTT: activated partial thromboplastin time, ANA: antinuclear antibodies, ANCA: antineutrophil cytoplasmic antibodies, ENA: extractable nuclear antigen, HIV: human immunodeficiency virus, CCP: cyclic citrullinated peptide antibody, G6PD: glucose 6 phosphate dehydrogenase, DNA: deoxyribonucleic acid.

Test	Day 1
Blood film	Polychromasia Increase in spherocytes
COVID-19 PCR	Negative
CRP	13
LDH	1790
Direct antiglobulin test	Positive
EBV IgG	Negative
EBV IgM	Negative
CMV IgG	Negative
CMV IgM	Negative
Urinary hemosiderin	Detected
Mycoplasma IgG	Negative
Mycoplasma IgM	Negative
INR	0.9
APTT	20
Fibrinogen	3.4
Hepatitis C antibody	Not detected
Hepatitis B surface antigen	Not detected
Hepatitis B core antibody	Not detected
Hepatitis A IgG antibody	Not detected
Haptoglobin	<0.08
ANA	320 speckled
Anti-DNA	<7
C-ANCA	<40
P-ANCA	<40
ENA Ab	Not detected
Blood group	B Pos/Anti C
Rheumatoid factor	<20
HIV 1/2 Ag/Ab	Negative
CCP antibody	<1
G6PD deficiency screen	Normal
Anti-DNA	<7

**Table 2:** Blood investigations H: abnormally high result, L: abnormally low result, ALP: alkaline phosphatase, GGT: gamma glutamyl transferase, ALT: alanine aminotransferase, HCT: hematocrit, MCV: mean corpuscular volume, WBC: white blood cell, NT: not tested.

	Day 0	Day 1	Day 1 (repeat)	Day 2	Day 3	Day 4	Day 5	Day 6	Day 6 (repeat)	Day 7	Day 8	Units
Sodium		139	132 (L)	135	134 (L)	137	136	136		135	138	mmol/L
Potassium		4.5	5	4.4	4.3	4.1	3.9	3.9		4.2	4.3	mmol/L
Chloride		98	100	104	104	107	106	106		108	109	mmol/L
Urea		4.2	7.5	5.9	7	7	6.9	7		8.1	11.0 (H)	mmol/L
Creatinine		84	74	63	73	80	76	82		79	81	umol/L
eGFR		80 (L)	85 (L)	>90	86 (L)	83 (L)	84 (L)	80 (L)		83(L)	81 (L)	mL/min/1.73 m^2^
Bicarbonate		24	23	22	22	22	22	23		21	21	mmol/L
Total bilirubin		6	82 (H)	65 (H)			61 (H)	66 (H)		56 (H)	97 (H)	umol/L
ALP		66	74	64			63	64		63	74	U/L
GGT		28	29	26			24	24		23	24	U/L
ALT		23	27	25			21	22		21	21	U/L
Total protein		68	69	60 (L)			57 (L)	59 (L)		58 (L)	64	g/L
Albumin		40	42	37	37	36	36	37		35	38	g/L
Globulin		28	27	23 (L)			21 (L)	22 (L)		23 (L)	26	g/L
Total calcium				2.25	2.26	2.17		2.23		2.17	2.27	mmol/L
Corrected calcium				2.31	2.32	2.25		2.29		2.27	2.31	mmol/L
Phosphate				1.03	1.27	1.3		1.3		1.35	1.35	mmol/L
Magnesium				0.79	0.81	2.02 (H)		0.89		1.10(H)	0.98	mmol/L
Conjugated bilirubin			9 (H)	12 (H)			10 (H)	10 (H)				umol/L
Uric acid						0.24						mmol/L
Lipase		19										U/L
Hemoglobin	106	96	82	78	72	65	58		74	NT	NT	g/L
HCT	0.32	0.29	0.24	0.23	0.22	0.2	0.19		0.23	NT	NT	L/L
MCV	96	99	95	97	104	107	110		108	NT	NT	/nL
WBC	7.6	7	6.7	8.4	7	14.8	12.7		13.8	NT	NT	/nL
Neutrophils	5.7	4.9	5.9	6.4	5.2	12.5	11		11.8	NT	NT	/nL
Lymphocytes	0.9	1	0.5	1	0.8	1.1	0.8		1	NT	NT	/nL
Monocytes	0.9	0.8	0.3	0.9	0.8	1.2	1		0.9	NT	NT	/nL
Eosinophils	0.2	0.2	<0.1	0.1	0.1	<0.1	<0.1		<0.1	NT	NT	/nL
Basophils	<0.1	<0.1	<0.1	<0.1	<0.1	<0.1	<0.1		<0.1	NT	NT	/nL
Platelets	257	264	229	248	277	302	291		264	NT	NT	/nL
Reticulocytes		186	166		234	246	255			NT	NT	/nL

**Figure 1:**
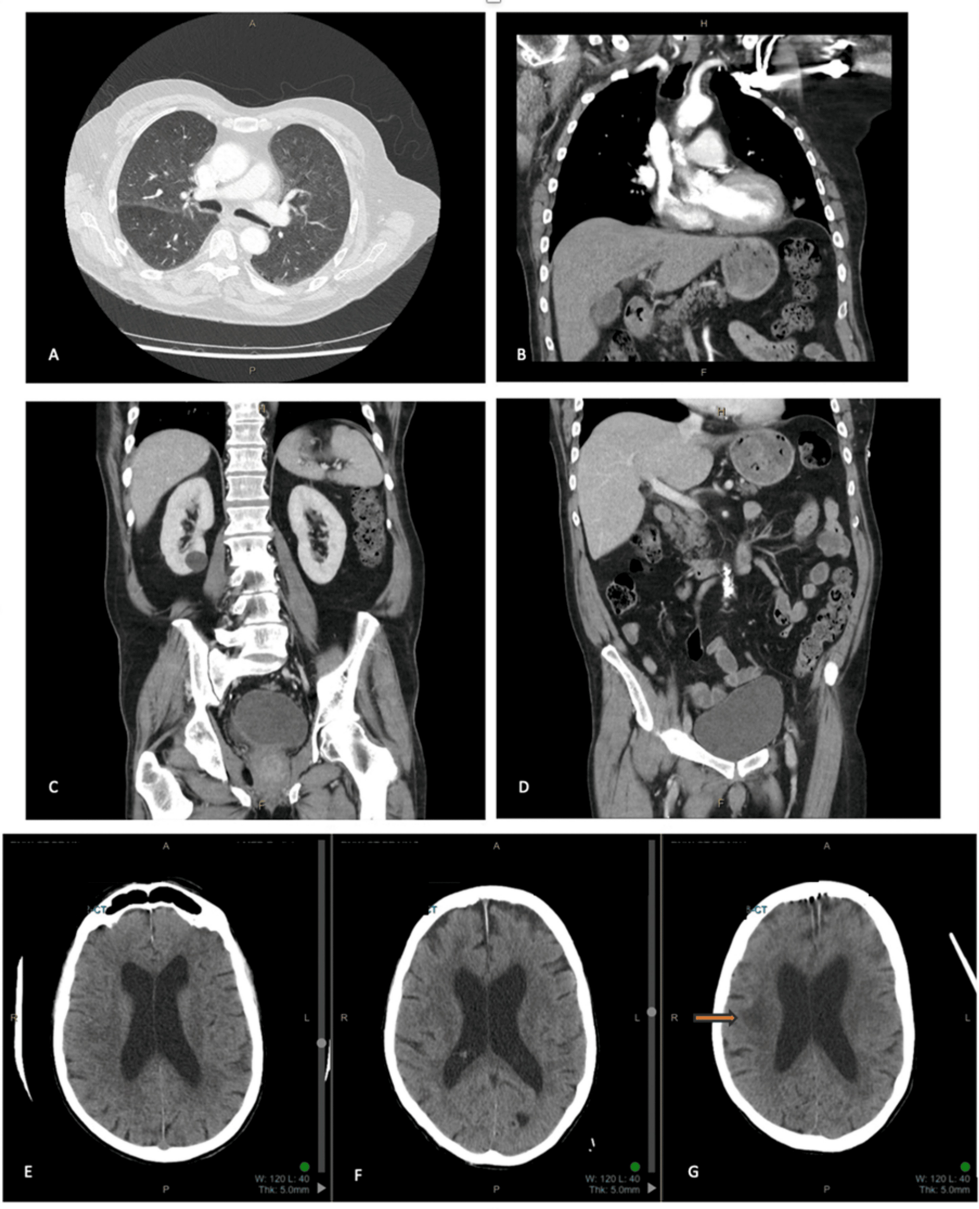
Contrast computed tomography (CT) of the chest: axial (A), coronal (B). Normal study. (C, D) Contrast CT of the abdomen and pelvis, coronal. Normal study. Non-contrast CT of the brain: day 1 (E), day 3 (F), day 4 (G). Disease progression with developing right-sided periventricular hypodensities (arrow)

Further investigation for underlying causes of AIHA, including viral serology, lymphoproliferative disorders, and autoimmune conditions, was unremarkable (Table 2). A CT scan of the chest, abdomen, and pelvis showed no evidence of malignancy (Figures 1A-1D). A non-contrast CT of the brain excluded acute infarction and hemorrhage (Figures 1E-1G).

The patient was commenced on high-dose oral prednisolone (100 mg daily) as per hematology opinion and prophylactic acyclovir and trimethoprim/sulfamethoxazole to prevent viral and opportunistic infections.

On day 3, he developed new neurological symptoms, including dysphagia, confusion, and worsening left-sided weakness. Glasgow Coma Scale (GCS) fluctuated from E4M6V2 (eye opening to voice, motor response localized to pain, incomprehensible verbal response) to GCS 15 (fully alert) and later declined to GCS 12, indicating moderate impairment of consciousness. A repeat non-contrast CT of the brain revealed white matter edema in the right parietal region without evidence of acute hemorrhage or infarction (Figure 1).

Urgent MRI was requested but was delayed due to technical issues and the patient's inability to tolerate the procedure prevented timely imaging. The MRI had to be canceled twice despite sedation, requiring anesthetic assistance, which further contributed to the delay.

Despite steroid treatment, hemoglobin levels did not improve, and transfusion was considered too risky due to the potential for worsening hemolysis. The patient was transferred to the ICU due to fluctuating GCS, where his neurological condition deteriorated, with marked left-sided weakness (2/5 strength in both upper and lower limbs). A single unit of blood was transfused due to worsening symptoms.

**Figure 2:**
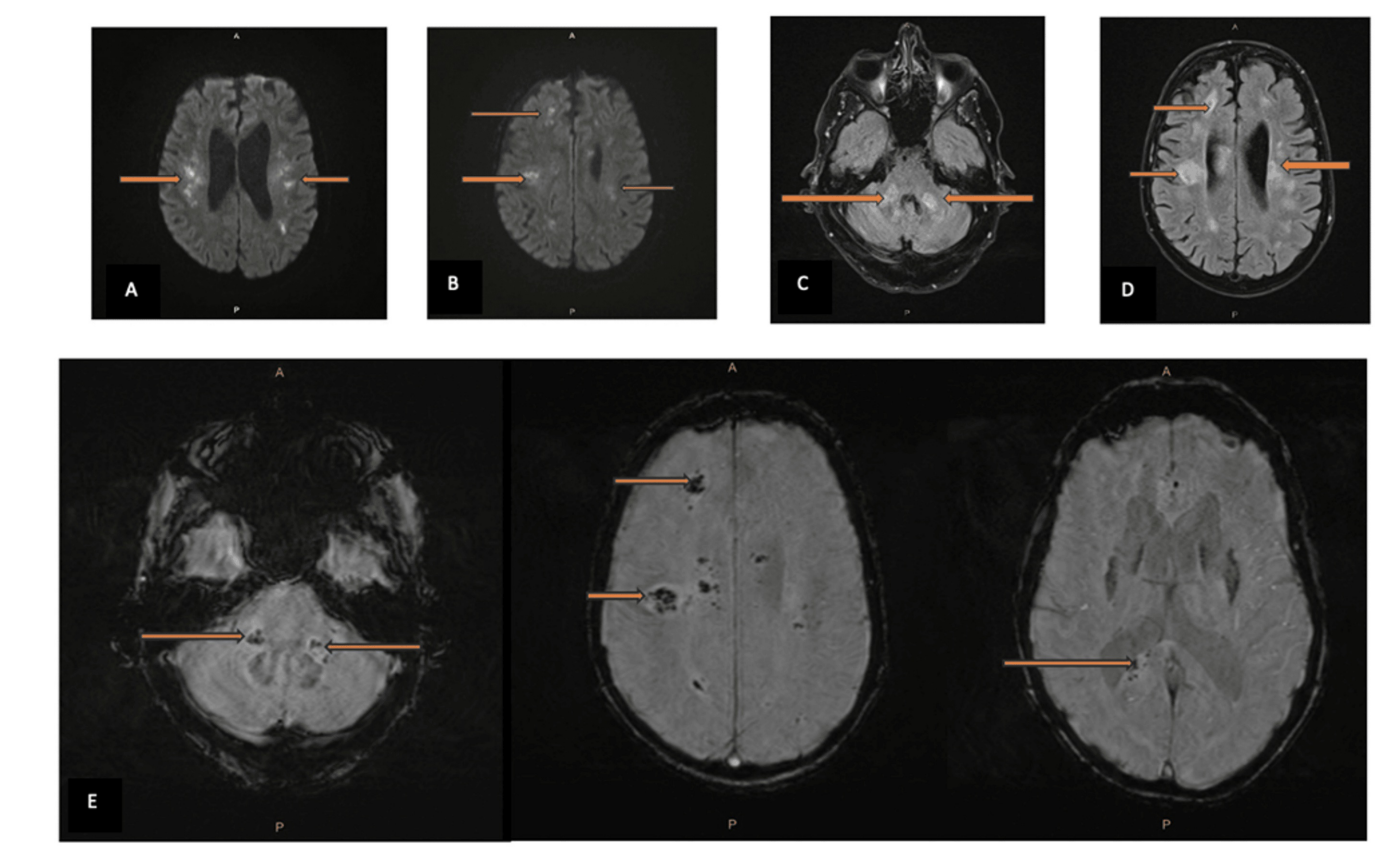
(A, B) Brain MRI diffusion-weighted images (DWIs): high SI denoting true restricted diffusion (arrows). (C, D) Brain MRI T2 FLAIR: showing high T2 and FLAIR signal distribution in cerebral hemispheres and white matter of the posterior fossa in the cerebral peduncles (arrows). (E) Brain MRI susceptibility-weighted images (SWIs): showing microhemorrhages (arrows). SI: signal intensity, FLAIR: fluid-attenuated inversion recovery.

On day 5, contrast-enhanced MRI revealed multifocal areas of hemorrhagic and ischemic lesions in both supra- and infratentorial white matter, consistent with leukoencephalopathy and vasculitis (Figure 2). Given the recent vaccinations, a diagnosis of hemorrhagic ADEM in the setting of AIHA was considered.

After extensive discussions with the family regarding the potential benefits and risks of diagnostic lumbar puncture and invasive treatments, the family opted for palliative care on day 6, and the patient declined further interventions. The patient passed away on day 8 post-admission. Consent for publication of this case was obtained from the patient's next of kin.

## Discussion

This case describes a rare and severe manifestation of concurrent AIHA and suspected hemorrhagic ADEM following recent varicella and influenza vaccinations in a 77-year-old male patient.

AIHA is characterized by premature red blood cell destruction via autoantibody mechanisms, often classified into warm and cold types based on temperature reactivity, with warm AIHA being predominantly mediated by IgG autoantibodies, causing extravascular hemolysis [1-4]. Our patient demonstrated classical laboratory features of warm AIHA, including normocytic anemia with a progressive hemoglobin drop, elevated lactate dehydrogenase (1790 U/L), indirect hyperbilirubinemia, low haptoglobin, a positive direct antiglobulin test (IgG only), and spherocytosis on peripheral smear, aligning with features described in previous literature [3,4]. Extensive investigations ruled out secondary causes such as lymphoproliferative disorders, infections, and autoimmune diseases, supporting a likely vaccine-associated trigger [1,2,4-8]. AIHA following vaccination is rare but has been reported, particularly with SARS-CoV-2 and influenza vaccines [3,7,8].

Simultaneously, the patient’s evolving neurological symptoms, including dysphasia, fluctuating consciousness, and left-sided weakness, raised suspicion for a CNS event. MRI findings revealed multifocal bilateral white matter lesions with hemorrhagic foci, consistent with AHLE, a severe variant of ADEM [1,2,5,6,9,10]. ADEM typically presents with encephalopathy, motor deficits, and multifocal neurological signs, and MRI usually shows asymmetric hyperintense lesions on T2 and FLAIR sequences, frequently involving deep white matter and brainstem structures [5,6,9]. Our patient's imaging findings, including restricted diffusion on DWI and microhemorrhages on SWI sequences, closely matched descriptions of severe hemorrhagic ADEM [9,10].

Differential diagnoses such as ischemic stroke, infectious meningoencephalitis, multiple sclerosis, neuromyelitis optica spectrum disorder, vasculitis, and progressive multifocal leukoencephalopathy were systematically considered and ruled out based on clinical presentation, negative infectious and autoimmune panels, and imaging features, although the absence of vascular imaging and lumbar puncture constituted limitations in confirming vasculitic processes [5,6]. The overall pattern of presentation was most consistent with hemorrhagic ADEM in the context of AIHA.

This case has limitations that warrant acknowledgment. Lumbar puncture was not performed due to patient instability and family preference, which limited the ability to confirm CNS inflammation or exclude infectious etiologies. Additionally, vascular imaging was not conducted, restricting the evaluation for vasculitis or thromboembolic processes. Delays in obtaining an MRI, although unavoidable, reduced the window for early neurological intervention. These factors may influence the certainty of diagnosis and should be considered when interpreting the findings.

To our knowledge, this represents one of the first reported cases of concurrent AIHA and hemorrhagic ADEM following sequential varicella and influenza vaccinations in an elderly patient, emphasizing the uniqueness and clinical significance of this immune response.

The temporal association between dual vaccination and the onset of dual autoimmune syndromes, in the absence of alternative etiologies, supports probable vaccine-induced immune dysregulation. Mechanistically, molecular mimicry, bystander activation, and polyclonal B-cell activation have been proposed as triggers for vaccine-associated autoimmune phenomena [1,3]. Immune senescence in the elderly, characterized by impaired regulatory T-cell function, may further predispose to exaggerated immune responses [4].

This case highlights the importance of early recognition of vaccine-associated autoimmune syndromes, particularly in elderly populations, and emphasizes the need for multidisciplinary management and awareness of potential rare but serious adverse immune events following immunization.

Further studies are needed to explore genetic predispositions, long-term outcomes, and effective treatment strategies for complex post-vaccination autoimmune clusters.

## Conclusions

The simultaneous occurrence of AIHA and suspected hemorrhagic ADEM following vaccination is exceptionally rare and highlights the potential for vaccine-related autoimmune complications, particularly in the elderly.

This case emphasizes the need for heightened clinical awareness of post-vaccination autoimmune manifestations, especially in elderly individuals who may be more susceptible to immune dysregulation and exaggerated responses. Future research should aim to elucidate the genetic and immunological mechanisms underlying complex autoimmune clustering and assess effective treatment strategies for such challenging presentations.

## References

[1] Baxter R, Lewis E, Goddard K (2016). Acute demyelinating events following vaccines: a case-centered analysis. Clin Infect Dis.

[2] Gaillard Gaillard, F. (2008 (2008). Acute disseminated encephalomyelitis (ADEM). Radiopaedia.org [Preprint].10.53347/rid-852. [6.

[3] Jacobs JW, Booth GS, Guarente J, Schlafer D, Zheng L, Adkins BD (2023). Autoimmune haemolytic anaemia and immune thrombocytopenia following SARS-CoV-2 and non-SARS-CoV-2 vaccination: 32 years of passive surveillance data. Br J Haematol.

[4] Jafarzadeh A, Jafarzadeh S, Pardehshenas M, Nemati M, Mortazavi SM (2023). Development and exacerbation of autoimmune hemolytic anemia following COVID-19 vaccination: a systematic review. Int J Lab Hematol.

[5] Kamr WH, El-Tantawy AM, Moustafa M, Abd-Elsalam OA (2017). ‘Acute disseminated encephalomyelitis: MR diffusion weighted imaging: Potential diagnostic value and outcome predilection. Egypt J Radiol Nucl Med.

[6] Koelman DL, Chahin S, Mar SS (2016). Acute disseminated encephalomyelitis in 228 patients: a retrospective, multicenter US study. Neurology.

[7] Mahdi N, Abdelmalik PA, Curtis M, Bar B (2015). A case of acute disseminated encephalomyelitis in a middle-aged adult. Case Rep Neurol Med.

[8] Montagnani S, Tuccori M, Lombardo G, Testi A, Mantarro S, Ruggiero E, Blandizzi C (2011). Autoimmune hemolytic anemia following MF59-adjuvanted influenza vaccine administration: a report of two cases. Ann Pharmacother.

[9] Schwarz S, Mohr A, Knauth M, Wildemann B, Storch-Hagenlocher B (2001). Acute disseminated encephalomyelitis: a follow-up study of 40 adult patients. Neurology.

[10] Sharma Sharma, R. and Elgendy, A. (2014) ‘Acute (2014). Acute hemorrhagic leukoencephalitis. Radiopaedia.org [Preprint]. 10.53347/rid-27630. [18.

